# Inhibition of Autophagy Promotes Salinomycin-Induced Apoptosis via Reactive Oxygen Species-Mediated PI3K/AKT/mTOR and ERK/p38 MAPK-Dependent Signaling in Human Prostate Cancer Cells

**DOI:** 10.3390/ijms18051088

**Published:** 2017-05-18

**Authors:** Kwang-Youn Kim, Kwang-Il Park, Sang-Hun Kim, Sun-Nyoung Yu, Sul-Gi Park, Young Woo Kim, Young-Kyo Seo, Jin-Yeul Ma, Soon-Cheol Ahn

**Affiliations:** 1 Department of Herbal Formula, Medical Research Center (MRC-GHF), College of Oriental Medicine, Daegu Haany University, Gyeongsan 38610, Korea; lokyve@dhu.ac.kr (K.-Y.K.); ywkim@dhu.ac.kr (Y.W.K.); 2 Department of Microbiology & Immunology, Pusan National University School of Medicine, Yangsan 50612, Korea; kshphone@nate.com (S.-H.K.); gurisn@naver.com (S.-N.Y.); betoje0728@gmail.com (S.-G.P.); 3 Korean Medicine (KM)-Application Center, Korea Institute of Oriental Medicine (KIOM), Daegu 41062, Korea; kipark@kiom.re.kr (K.-I.P.); jyma@kiom.re.kr (J.-Y.M.); 4 School of Life Sciences, Ulsan National Institute of Science and Technology, Ulsan 44919, Korea; ykseo@unist.ac.kr; 5 Immunoregulatory Therapeutics Group in Brain Busan 21 Project, Pusan National University, Yangsan 50612, Korea

**Keywords:** salinomycin, reactive oxygen species, apoptosis, autophagy, prostate cancer cells

## Abstract

Recently, the interplay between autophagy and apoptosis has become an important factor in chemotherapy for cancer treatment. Inhibition of autophagy may be an effective strategy to improve the treatment of chemo-resistant cancer by consistent exposure to chemotherapeutic drugs. However, no reports have clearly elucidated the underlying mechanisms. Therefore, in this study, we assessed whether salinomycin, a promising anticancer drug, induces apoptosis and elucidated potential antitumor mechanisms in chemo-resistant prostate cancer cells. Cell viability assay, Western blot, annexin V/propidium iodide assay, acridine orange (AO) staining, caspase-3 activity assay, reactive oxygen species (ROS) production, and mitochondrial membrane potential were assayed. Our data showed that salinomycin alters the sensitivity of prostate cancer cells to autophagy. Pretreatment with 3-methyladenine (3-MA), an autophagy inhibitor, enhanced the salinomycin-induced apoptosis. Notably, salinomycin decreased phosphorylated of AKT and phosphorylated mammalian target of rapamycin (mTOR) in prostate cancer cells. Pretreatment with LY294002, an autophagy and PI3K inhibitor, enhanced the salinomycin-induced apoptosis by decreasing the AKT and mTOR activities and suppressing autophagy. However, pretreatment with PD98059 and SB203580, an extracellular signal-regulated kinases (ERK), and p38 inhibitors, suppressed the salinomycin-induced autophagy by reversing the upregulation of ERK and p38. In addition, pretreatment with *N*-acetyl-l-cysteine (NAC), an antioxidant, inhibited salinomycin-induced autophagy by suppressing ROS production. Our results suggested that salinomycin induces apoptosis, which was related to ROS-mediated autophagy through regulation of the PI3K/AKT/mTOR and ERK/p38 MAPK signaling pathways.

## 1. Introduction

Prostate cancer is the most frequently diagnosed and the most fatal type of cancer in men [1]. Recently, prostate cancer incidence and mortality rates have rapidly increased in most Asian countries, including Korea and Japan [2]. Many investigators have attempted to identify the underlying mechanisms of these cancers, but these are still unknown. Recently, several new therapies have been tested against prostate cancer, including vaccines, monoclonal antibodies, and other types of targeted drugs [3]. Importantly, new drugs and combination therapies have been used to target chemo-resistant and advanced metastatic prostate cancer [4].

Autophagy is a new target mechanism for chemo-resistant cancer therapy [5]. Autophagy is a catabolic mechanism that involves degradation of unnecessary or dysfunctional components [6]. This process enables cells to cope with various stresses, such as nutrient deprivation, endoplasmic reticulum (ER) stress, pathogen infection, or hypoxia, which is considered a survival mechanism [7]. However, during harsh or the prolonged stress or in apoptosis-deficient cells, autophagy can participate in cell death [8]. More recently, it was reported that reactive oxygen species (ROS) are key molecules in crosstalk between apoptosis and autophagy [9,10]. ROS regulate cell survival and growth, but excessive ROS lead to irreversible cellular damage, such as autophagy and/or apoptosis induction in several types of cancer cells [11]. Additionally, these two processes share many pathways commonly used in the response to diverse stressors. For example, ROS overproduction as well as phosphatidylinositol 3-kinase/Akt (a serine/threonine kinase also known as protein kinase B (PKB))/mammalian target of rapamycin (PI3K/AKT/mTOR) inactivation and mitogen-activated protein kinase (MAPK) activation have been reported in both apoptosis and autophagy [12,13].

Salinomycin is a monocarboxylic ionophore isolated from *Streptomyces albus* [14]. It has been used to transport cations across biological membranes as a novel class of anti-cancer agents [15,16]. We previously reported that salinomycin induces apoptosis via cell cycle arrest and ROS-mediated mitochondrial pathways in prostate cancer cells [9,17]. Additionally, we demonstrated that this drug enhanced cytotoxicity by decreasing the efflux of doxorubicin in multidrug-resistant breast cancer cells [18]. More recently, salinomycin was shown to induce autophagy with concomitant ROS production or ER stress in human cancer cells [19].

The present study investigated the autophagy pathway as a protective factor of apoptosis in chemo-resistant prostate cancer cells. With the aim of exploring the anticancer effects of salinomycin, we examined the induction of apoptosis and autophagy by salinomycin in prostate cancer cells. Our results demonstrated that salinomycin inhibits the viability of cells by modulating apoptosis and autophagy. Inhibition of the autophagy pathway enhanced the salinomycin-induced apoptosis, which confirmed the role of ROS in crosstalk between autophagy and apoptosis. Thus, autophagy inhibition may be a therapeutic strategy to improve the treatment of chemo-resistant cancer.

## 2. Results

### 2.1. Salinomycin Induces Both Apoptosis and Autphagy in Human Prostate Cancer Cells

**Figure 1:**
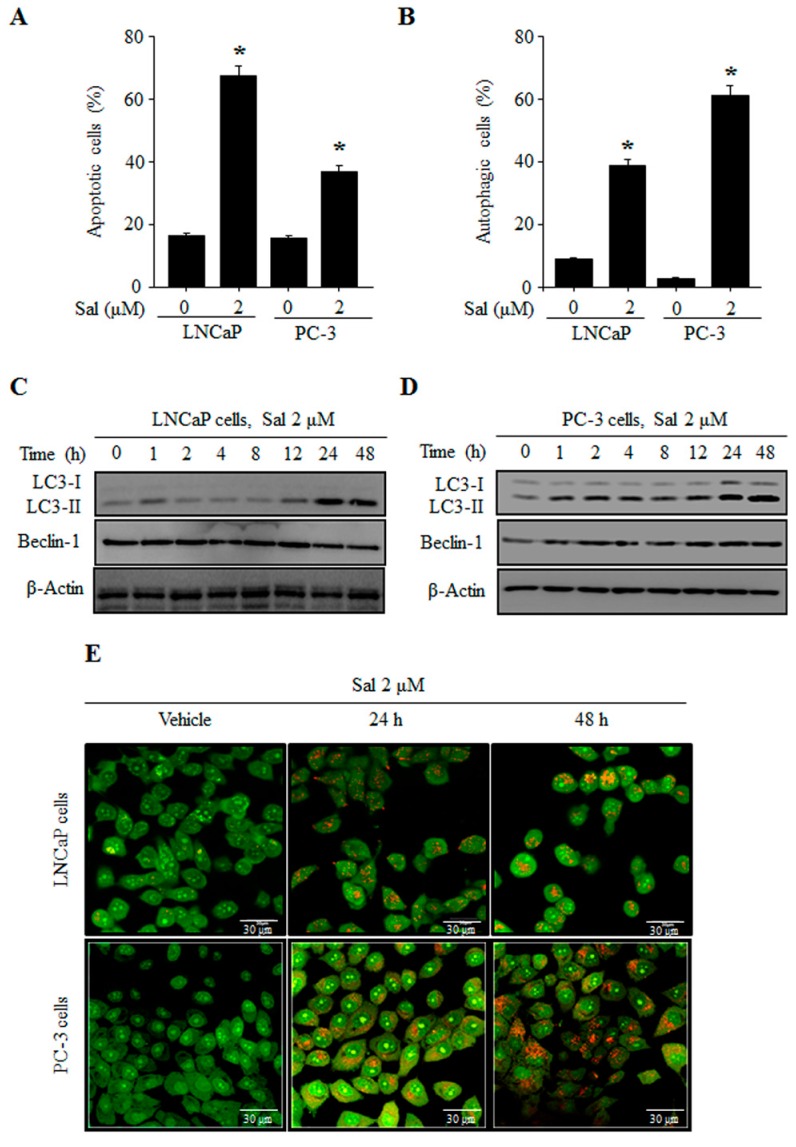
Salinomycin (Sal) induces both apoptosis and autophagy in human prostate cancer cells. (**A**) Apoptosis induction. Flow cytometric analysis of annexin-V/propidium iodide (PI) staining; and (**B**) autophagy induction. Flow cytometric analysis of acridine orange (AO) staining. LNCaP and PC-3 cells were treated with 2 µM salinomycin for 48 h; (**C**,**D**) LC3 and Beclin-1 expression; (**C**) LNCaP cells; and (**D**) PC-3 cells. Cells were treated with 2 µM salinomycin for the indicated times. After treatment, total cell lysates were subjected to SDS-PAGE for Western blot analysis; and (**E**) acidic vesicular detection. After treatment with 2 µM salinomycin for the indicated times, cells were stained with AO, and then cells were visualized using a laser scanning confocal microscope. Magnification, 600×. All data were representative of at least three independent experiments. Data were shown as the mean ± SD. * *p* < 0.05.

Previously, we reported the cytotoxic effects of salinomycin on human prostate cancer cell lines using MTT assays [9]. Additionally, we confirmed the salinomycin induced apoptosis in human prostate cancer PC-3 cells. However, the resistance to salinomycin-induced apoptosis between the PC-3 and LNCaP cells is unknown. Consistent with previous results, we confirmed that LNCaP cells were more susceptible to salinomycin than PC-3 cells. Furthermore, non-malignant RWPE-1 cells were relatively less sensitive to salinomycin, which did not significantly inhibit cell viability (data not shown). The percentages of apoptotic cells were 67.09% and 34.75% at 2 µM salinomycin in LNCaP and PC-3 cells, respectively. These results indicated that LNCaP cells were more sensitive to apoptosis than PC-3 cells (Figure 1A).

Next, to determine whether salinomycin decreases cell survival via induction of autophagy, we verified the autophagy markers in salinomycin-treated prostate cancer cells using acridine orange (AO) staining. Salinomycin induced the accumulation of acidic vesicles (Figure 1B). Based on a comparison of the acidic vesicular rate, PC-3 cells had a higher susceptibility to autophagy than that of LNCaP cells, which was the opposite of the apoptosis results (Figure 1B). Additionally, LC3-I/II activated forms and Beclin-1 expression levels were increased in PC-3 cells compared to those of LNCaP cells in a time-dependent manner (Figure 1C,D). The formation of acidic vesicular organelles was assessed using confocal microscopy, control cells displayed green fluorescence with minimal red fluorescence, while salinomycin-treated LNCaP and PC-3 cells showed an increase in red fluorescence (Figure 1E). Consequently, we hypothesized that the differences in salinomycin-induced cytotoxicity between chemo-sensitive LNCaP cells and chemo-resistant PC-3 cells were associated with the induction of autophagy.

### 2.2. Autophagy Inhibition Enhances Salinomycin-Induced Apoptosis in Prostate Cancer Cells

**Figure 2:**
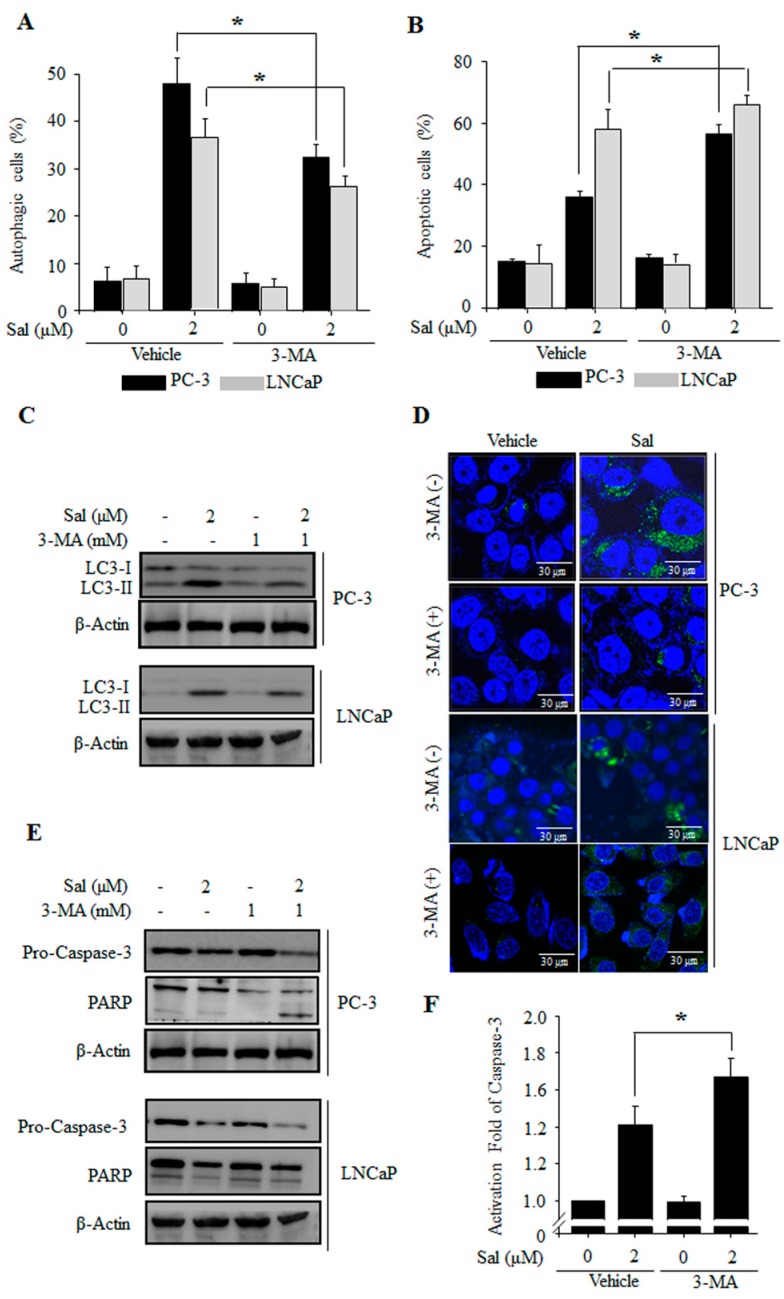
Autophagy inhibition enhances salinomycin-induced apoptosis in prostate cancer cells. (**A**) The effects of 3-MA on salinomycin-induced autophagy. Cells were pretreated with 1 mM 3-MA for 1 h, and acridine orange (AO) stained cells were evaluated by flow cytometry; (**B**) the effects of 3-MA on salinomycin-induced apoptosis. Cells were pretreated with 1 mM 3-MA for 1 h, annexin-V-FITC stained cells were evaluated by flow cytometry; (**C**) the effects of 3-MA on LC3, caspase-3 and PARP protein expression. Cells were pretreated with 1 mM 3-MA for 1 h, and the proteins were detected by Western blot analysis; (**D**) the effects of 3-MA on immunofluorescence staining for LC3. Cells were pretreated with 1 mM 3-MA for 1 h, and the fluorescence was detected by confocal microscopy. Magnification, 1800×; (**E**) the effects of 3-MA on LC3, caspase-3 and PARP protein expression. Cells were pretreated with 1 mM 3-MA for 1 h, and the proteins were detected by western blot analysis; and (**F**) the effect of 3-MA on caspase-3 activity. Cells were pretreated with 1 mM 3-MA for 1 h, and the proteins were detected by a caspase-3 colorimetric assay kit. All data were representative of at least three independent experiments. Data were shown as the mean ± SD. * *p* < 0.05.

To further confirm the involvement of autophagy in salinomycin-induced apoptosis, we evaluated the effects of 3-methyladenine (3-MA), an autophagy inhibitor. Salinomycin-induced autophagy was significantly inhibited by 3-MA in both PC-3 and LNCaP cells (Figure 2A). Furthermore, salinomycin-induced apoptosis were enhanced by autophagy inhibition (Figure 2B). Additionally, we confirmed by Western blot and confocal microscopy analysis that the appearance of punctate LC-3B dots was inhibited in 3-MA pretreated cells (Figure 2C,D). Additionally, salinomycin-induced caspase-3 activation and poly (ADP-ribose) polymerase (PARP) protein cleavage were enhanced by autophagy inhibition (Figure 2E,F). Taken together, the results indicated that salinomycin-induced autophagy protects against apoptosis in prostate cancer cells and inhibition of autophagy subsequently enhances apoptosis through caspase activation in prostate cancer cells.

### 2.3. The PI3K/AKT/mTOR Pathway Is Involved in the Regulation of Both Salinomycin-Induced Autophagy and Apoptosis in Prostate Cancer Cells

**Figure 3:**
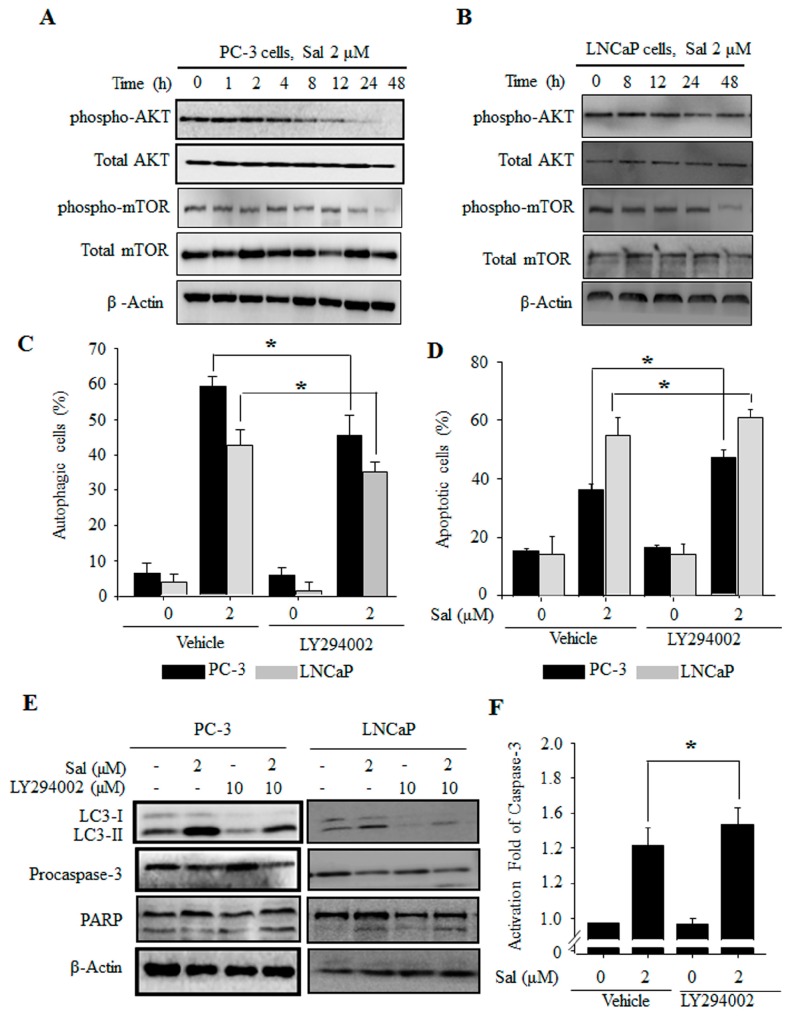
The PI3K/AKT/mTOR pathway is involved in the regulation of both salinomycin-induced autophagy and apoptosis in prostate cancer cells. (**A**,**B**) AKT/mTOR expression. (**A**) PC-3 cells. (**B**) LNCaP cells. Cells were treated with 2 µM salinomycin for the indicated times. After treatment, total cell lysates were subjected to SDS-PAGE for Western blot analysis; (**C**) the effects of LY294002 on salinomycin-induced autophagy. Cells were pretreated with 10 µM LY294002 for 1 h, and acridine orange (AO) stained cells were evaluated by flow cytometry; (**D**) the effects of LY294002 on salinomycin-induced apoptosis. Cells were pretreated with 10 µM LY294002 for 1 h, and annexin-V-FITC stained cells were evaluated by flow cytometry; (**E**) the Effects of LY294002 on LC3, procaspase-3 and PARP protein expression. Cells were pretreated with 10 µM LY294002 for 1 h, and then the proteins were detected by Western blot analysis; and (**F**) the effect of LY294002 on caspase-3 activity. PC-3 cells were pretreated with 10 µM LY294002 for 1 h, and the proteins were detected by a caspase-3 colorimetric assay kit. All data were representative of at least three independent experiments. Data were shown as the mean ± SD. * *p* < 0.05.

To ascertain the possible role of PI3K/AKT/mTOR pathways in salinomycin-induced cell death, we verified the expression levels of the phosphorylated forms of AKT and mTOR. The expression of phosphorylated AKT was decreased in a time-dependent manner in both PC-3 and LNCaP cells (Figure 3A,B). Furthermore, mTOR, an important downstream executor of AKT, showed decreased phosphorylation (Figure 3A,B). Next, we determined the effects of the PI3K/AKT pathway using AO staining. Salinomycin-induced autophagy was recovered by LY294002, a PI3K/AKT inhibitor in both PC-3 and LNCaP cells (Figure 3C). Additionally, protein expression of the autophagy marker LC-3 was rescued (Figure 3E). Furthermore, the inhibition of autophagy by LY294002 enhanced salinomycin-induced apoptosis (Figure 3D), which was associated with caspase-3 activation (Figure 3E,F) and PARP cleavage (Figure 3E). Inhibition of autophagy by 3-MA treatment enhanced inactivation of PI3/AKT/mTOR (data not shown). These data showed that inhibition of autophagy by LY294002 and 3-MA enhanced salinomycin-induced apoptosis. Therefore, the PI3K/AKT/mTOR pathway is involved in the regulation of both autophagy and apoptosis induced by salinomycin.

### 2.4. The ERK and p38 MAPK Pathways Are Involved in the Regulation of Salinomycin-Induced Autophagy in Prostate Cancer Cells

**Figure 4:**
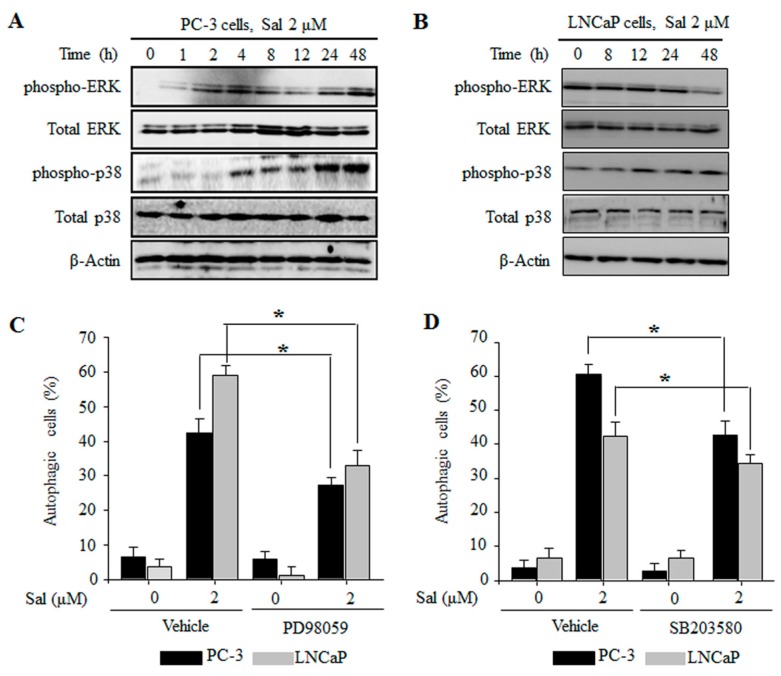
The ERK and p38 MAPK pathways are involved in the regulation of salinomycin-induced autophagy in prostate cancer cells. (**A**,**B**) ERK and p38 MAPK protein expression. (**A**) PC-3 cells. (**B**) LNCaP cells. Cells were treated with 2 µM salinomycin for the indicated times. After treatment, total cell lysates were subjected to SDS-PAGE for Western blot analysis; (**C**) the effect of PD98059 on salinomycin-induced autophagy; (**D**) the effect of SB203580 on salinomycin-induced autophagy. Cells were pretreated with 10 µM PD98059 or 10 µM SB203580 for 1 h, and acridine orange (AO) stained cells were evaluated by flow cytometry. All data were representative of at least three independent experiments. Data were shown as the mean ± SD. * *p* < 0.05.

Several reports have been shown that MAPK signaling pathways are involved in the regulation of autophagy [12]. We found that salinomycin activated extracellular signal-regulated kinases (ERK) and p38 MAPK in a time-dependent manner in both PC-3 and LNCaP cells (Figure 4A,B). However, JNK expression was not changed (data not shown). To further confirm whether regulation of the MAPK pathway affects salinomycin-induced autophagy in prostate cancer cells, we used AO staining and Western blot analysis. Both PD98059, an ERK inhibitor, and SB203580, a p38 inhibitor, decreased salinomycin-induced autophagy in both PC-3 and LNCaP cells (Figure 4C,D). Additionally, protein expression of the autophagy marker LC3 was recovered (data not shown). These results showed that ERK and p38 MAPK activation are closely linked to salinomycin-induced autophagy in prostate cancer cells.

### 2.5. Autophagy Inhibition Stimulates ROS Production in Prostate Cancer Cells

**Figure 5:**
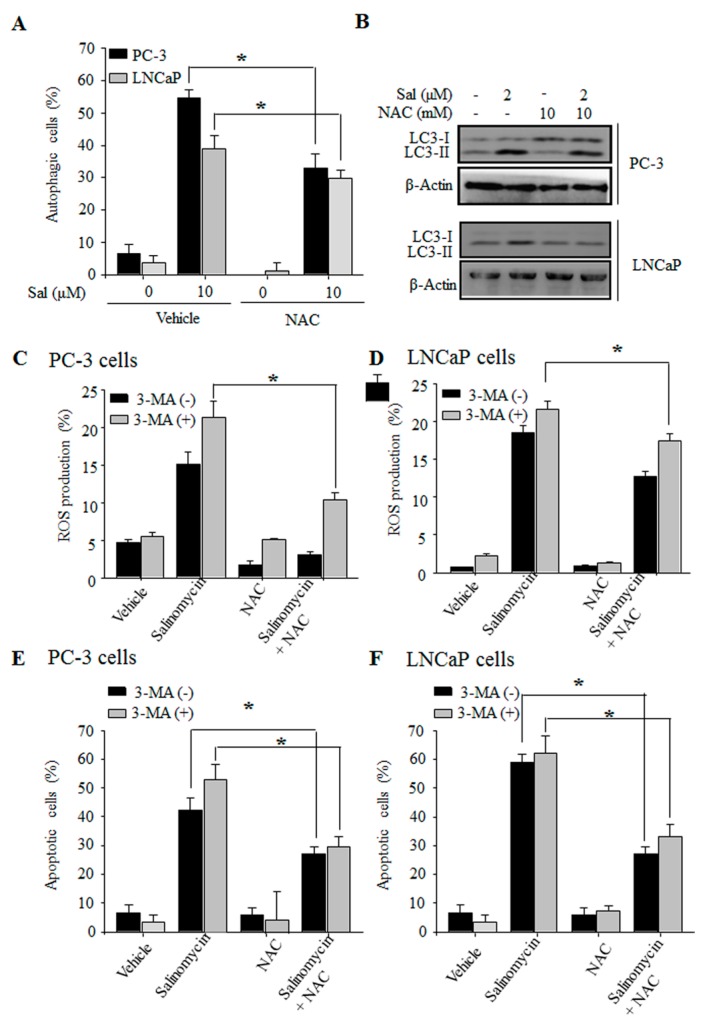
Autophagy inhibition stimulates ROS production in prostate cancer cells. (**A**) Effects of NAC on salinomycin-induced autophagy. Cells were pretreated with 10 mM NAC for 1 h, and acridine orange (AO) stained cells were evaluated by flow cytometry; (**B**) the effects of NAC on LC-3 protein expression. Cells were pretreated with 10 mM NAC for 1 h, and total cell lysates were subjected to SDS-PAGE forW blot analysis; (**C**,**D**) the effects of 3-MA on salinomycin-induced ROS production. (**C**) PC-3 cells; (**D**) LNCaP cells; and (**E**,**F**) the effects of 3-MA on ROS-mediated salinomycin-induced apoptosis. (**E**) PC-3 cells. (**F**) LNCaP cells. Cells were pretreated alone or with a combination of 1 mM 3-MA and 10 µM NAC for 1 h, and then annexin-V-FITC or DCF-DA stained cells were evaluated by flow cytometry. All data were representative of at least three independent experiments. Data were shown as the mean ± SD. * *p* < 0.05.

Previously, we reported that salinomycin induces apoptosis via accumulation of reactive oxygen species (ROS) and the mitochondrial pathway in PC-3 cells [9]. Several recent reports have shown that ROS are key molecules in apoptosis and autophagy [11]. We assessed whether ROS production is associated with salinomycin-induced autophagy of prostate cancer cells. Salinomycin-induced autophagy was recovered by *N*-acetyl-l-cysteine (NAC), a ROS scavenger in both PC-3 and LNCaP cells (Figure 5A). Consistent with AO staining results, salinomycin-induced LC3 conversion was inhibited by NAC in prostate cancer cells (Figure 5B). Next, to determine whether inhibition of autophagy stimulates ROS production, we analyzed the cells for ROS production by flow cytometry. ROS levels showed greater increases in the cells treated with a combination of salinomycin and 3-MA than that of salinomycin alone (Figure 5C,D). This increase was efficiently recovered when the cells were pretreated with NAC in both PC-3 and LNCaP cells (Figure 5C,D). Finally, the increase in salinomycin-induced apoptosis by 3-MA was completely prevented by the inhibition of ROS-mediated autophagy by NAC (Figure 5E,F). Furthermore, we assessed the effects of ROS production between the PI3K/AKT/mTOR and MAPK pathways. The results showed that the combination treatment with salinomycin and NAC increased AKT and mTOR phosphorylation compared with that of salinomycin treatment alone (Supplementary Figure S1A). ERK and p38 MAPK were decreased by the combination treatment with salinomycin and NAC (Supplementary Figure S1B). These results showed that ROS production plays a key role in both apoptosis and autophagy. In addition, ROS production is upstream of the PI3K/AKT/mTOR and MAPK pathways in salinomycin-induced autophagy.

## 3. Discussion

Salinomycin is an ionophore isolated from *Streptomyces albus*, which has been used as a monocarboxylic polyether antibiotic [14]. Many reports have shown that salinomycin has potent anticancer activity through the induction of apoptosis, which is dependent on various molecular mechanisms, such as upregulation of p21, downregulation of survivin, calpain, and cytochrome c-mediated cell death in many cancer cell lines [17,20]. Additionally, salinomycin markedly inhibited cell growth in a variety of cancer cells, including drug-sensitive cells and -resistant cancer cells [18,21]. Previous reports showed that salinomycin induced ROS-mediated apoptosis via mitochondrial dysfunction in PC-3 cells [9]. Here, we demonstrated that salinomycin induces ROS-mediated apoptosis in prostate cancer cells, accompanied by an increase in autophagy for cancer cell survival.

Interestingly, salinomycin has different effects of apoptosis depending on the cell type. In this study, we showed that the apoptotic rates of hormone-independent and chemo-resistant PC-3 cells were much lower than those of the hormone-dependent and chemo-sensitive LNCaP cells (Figure 1). These results suggested that other cytoprotective mechanisms are involved in apoptosis resistance, for example, autophagy induction by salinomycin. Autophagy is one of the key mechanisms to remove mis-folded or aggregated proteins and maintain energy homeostasis during the stress response [22,23]. Recently, autophagy has been widely studied in chemo-resistant cancer [24,25] and the regulators of crosstalk between apoptosis and autophagy have also been examined [11,26]. In this study, salinomycin induced autophagy by LC3 conversion in all of the cell lines. However, salinomycin-induced autophagy rates in drug-sensitive LNCaP cells were lower than those in drug-resistant PC-3 cells (Figure 1). Additionally, as expected, salinomycin-induced autophagy was inhibited by an autophagy inhibitor, 3-MA. Inhibition of autophagy enhanced salinomycin-induced apoptosis via caspase-3 activation in both PC-3 and LNCaP cells (Figure 2). These results indicated that autophagy affects the resistance to apoptosis in cancer cells.

Many reports have shown that the mechanisms linking apoptosis and autophagy are related to ROS production [10,27]. Recent evidence has demonstrated crosstalk between autophagy and apoptosis through several commonly molecules, such as p53, Bcl-2 families and PI3K/AKT/mTOR signal pathways [12]. Additionally, the PI3K/AKT/mTOR pathway plays an important role in the resistance to a number of anti-cancer agents [28]. Mitogen-activated protein kinase (MAPK) pathways such as C-jun N-terminal kinase (JNK), p38 and extracellular signal-regulated kinases (ERK), are involved in cell survival and resistance associated with autophagy and apoptosis in many cancer cells following exposure to different stresses [29]. Additionally, it has been reported that ROS inhibits PI3K/AKT/mTOR signaling [30] and induces MAPK signaling [31]. Furthermore, intracellular ROS production has vital roles in PI3K/AKT/mTOR inactivation and MAPK activation during apoptosis and autophagy [32]. In this study, autophagy and LC3 expression were significantly restored by a ROS scavenger, NAC, in both PC-3 and LNCaP cells. In addition, inhibition of autophagy led to an increase in ROS formation and the salinomycin-induced apoptosis in both PC-3 and LNCaP cells (Figure 5). Moreover, the inactivation of AKT/mTOR by the PI3K/AKT inhibitor LY294002 enhanced salinomycin-induced apoptosis (Figure 3). Inactivation of MAPK proteins ERK and p38 decreased salinomycin-induced autophagy by the ERK inhibitor PD98059 or the p38 MAPK inhibitor SB203580, respectively (Figure 4). Furthermore, inhibition of ROS generation linked to AKT/mTOR, ERK and p38 activation was restored in both PC-3 and LNCaP cells. These results implied that salinomycin-induced autophagy is triggered the ROS-mediated PI3K/AKT/mTOR and MAPK pathways.

## 4. Materials and Methods

### 4.1. Reagents and Antibodies

Acridine orange (AO), 2′,7′-dichlorodihydrofluorescein diacetate (DCF-DA), 4′,6-diamidino-2-phenylindole dihydrochloride (DAPI), *N*-acetyl-l-cysteine (NAC) and 3-methyladenine (3-MA) were purchased from Sigma Chemical Co. (St. Louis, MO, USA). A FITC Annexin V Apoptosis Detection Kit (BD Biosciences, San Jose, CA, USA) was obtained. A Caspase-3 colorimetric assay kit was obtained from R&D Systems Inc. (Minneapolis, MN, USA). LY294002, PD98059 and SB203580 were purchased from TOCRIS (Bristol, UK). The ECL Western Kit was purchased from Amersham (Arlington Heights, IL, USA). Antibodies against LC-3B, Beclin-1, β-actin, phosphor-AKT, total AKT, phosphor-ERK, total ERK, phospho-p38, total p38, phosphor-JNK, and total JNK were purchased from Cell Signaling Technology (Beverly, MS, USA) and Santa Cruz Biotechnology (Santa Cruz, CA, USA). The goat anti-mouse IgG and goat anti-rabbit secondary antibodies were purchased from Enzo Life Science (Farmingdale, NY, USA). The FITC-conjugated anti-rabbit IgG secondary antibody was purchased from BioFX Laboratories (Owings Mills, MD, USA).

### 4.2. Cell Lines and Cell Culture

The human prostate cancer cell lines PC-3 and LNCaP cells were obtained from the American Type Culture Collection (ATCC, Manassas, VA, USA). These cells were maintained and cultured in DMEM (WelGENE Inc., Daejeon, Korea) supplemented with 10% fetal bovine serum (FBS) (WelGENE Inc.), 100 units/mL of penicillin and 100 µg/mL of streptomycin (WelGENE Inc.) at 37 °C in a humidified atmosphere with 5% CO_2_.

### 4.3. Annexin V/Propidium Iodide (PI) Assay

For detection of apoptotic cells, a FITC Annexin V Apoptosis Detection Kit was used. Briefly, human prostate cancer PC-3 and LNCaP cells were cultured in 6-well plates at a density of 5 × 10^4^/well. After treatment with salinomycin, cells were harvested, and then mixed in 100 µL of 1× binding buffer and stained with annexin-V/propidium iodide (PI) at room temperature for 15 min. The stained cells were analyzed by flow cytometry (FACSCalibur, Becton Dickinson, Franklin Lakes, NJ, USA), and the apoptotic cells were calculated using Cell Quest Pro software (version 5.1) on Mac^®^ OS 9 (Becton Dickinson).

### 4.4. Detection of Acidic Vesicular Organelles

For analysis of the acidic vesicular organelles, human prostate cancer PC-3 and LNCaP cells were cultured in a glass-bottom dish. After treatment with salinomycin for the indicated times, cells were stained with 1 µM acridine orange at 37 °C in the dark for 20 min. Then cells were washed with phosphate-buffered saline (PBS) and visualized using with a laser scanning confocal microscope (Olympus FluoView FV1000, Tokyo, Japan). Additionally, to quantify the number of acidic vesicles, cells were harvested and washed with PBS. Then, the cells were analyzed by flow cytometry (FACSCalibur, Becton Dickinson) and calculated using Cell Quest Pro software (version 5.1) on Mac^®^ OS 9 (Becton Dickinson).

### 4.5. Immunofluorescence for LC-3

The LC-3 expression levels were determined using an immunofluorescence analysis. Human prostate cancer PC-3 and LNCaP cells were seeded in a glass-bottom dish. After treatment with salinomycin with or without 1 mM 3-MA for 48 h, the cells were fixed with 3% (*v*/*v*) paraformaldehyde for 10 min. After fixation, the cells were permeabilized with 0.5% (*v*/*v*) Triton X-100 for 10 min and blocked with 3% (*w*/*v*) bovine serum albumin (BSA) for 2 h at room temperature. After blocking, the cells were incubated with primary LC-3 antibody (1:400 diluted in BSA buffer) at room temperature for 2 h and then reacted with FITC-conjugated anti-rabbit IgG secondary antibody. DAPI was used to stain the nuclei. Samples were visualized with a laser scanning confocal microscope (Olympus FluoView FV1000).

### 4.6. Measurement of Intracellular ROS Generation

Intracellular ROS generation was measured using the DCF-DA fluorescent dye. Human prostate cancer PC-3 and LNCaP cells were cultured in six-well plates at a density of 5 × 10^4^/well. After treatment with salinomycin, the cells were incubated with 10 µM DCF-DA at 37 °C for 30 min. Cells were washed twice with PBS and analyzed with flow cytometry (FACSCalibur, Becton Dickinson) and Cell Quest Pro software (version 5.1) on Mac^®^ OS 9 (Becton Dickinson).

### 4.7. Western Blot Analysis

Cell extracts were prepared by incubating the cells in lysis buffer (150 mM NaCl, 10 mM Tris (pH 7.4), 5 mM EDTA (pH 8.0), 1% Triton X-100, 1 mM PMSF, 20 µg/mL aprotinin, 50 µg/mL leupeptin, 1 mM benzidine, 1 mg/mL pepstatin, 8 mM sodium pyrophosphate, and 20 mM β-glycerophosphate). Forty micrograms of proteins were electrophoretically separated using sodium dodecyl sulfate-polyacrylamide gel electrophoresis (SDS-PAGE) with an 8–15% gel and transferred to a polyvinylidenefluoride (PVDF) membrane. After blocking with TBS-T buffer (20 mM Tris (pH 7.4), 150 mM NaCl, and 0.1% Tween 20) containing 5% skim milk, membranes were incubated with primary and secondary antibodies. The membranes were then washed with TBS-T buffer and visualized with ECL Western blot analysis detection reagents. The density of each band was determined with a fluorescence scanner (LAS 3000, Fuji Film, Tokyo, Japan) and analyzed with Multi Gauge V3.0 software (Fuji Film).

### 4.8. Measurement of Caspase-3 Activity

For detection of caspase-3 activation, a caspase-3 colorimetric assay kit (R&D Systems Inc.) was used according to the manufacturer’s protocol. Equal amounts of protein (220 µg) were resuspended in reaction buffer containing substrate (Ac-DEVD-pNA) and then incubated at 37 °C for 4 h in the dark. The absorbance of the released pNA was measured at 405 nm using an ELISA reader.

### 4.9. Statistical Analysis

Experiments were repeated at least three times with consistent results. Unless otherwise stated, data were expressed as the mean ± SD. ANOVA was used to compare the experimental groups to the control values, whereas comparisons between multiple groups were performed using Tukey’s multiple comparison test. The results were statistically significant at * *p* < 0.05.

## 5. Conclusions

In conclusion, salinomycin markedly inhibited the cell growth in not only drug-sensitive cells, but also drug-resistant cells. Above all, salinomycin has different effects on apoptosis depending on the cell type. Additionally, ROS play a critical role in salinomycin-induced autophagy regulated via ROS-mediated PI3K/AKT/mTOR and ERK/p38 MAPK pathways in prostate cancer cells. Furthermore, the enhancement of apoptosis induced by ROS-mediated autophagy inhibition suggested that cross talk between apoptosis and autophagy functions as a resistance mechanism against apoptosis.

## Supplementary Materials

Supplementary materials can be found at www.mdpi.com/1422-0067/18/5/1088/s1.

## Abbreviations

3-MA	3-Methyladenine
AO	Acridine orange
AVOs	Acidic vesicular organelles
BSA	Bovine serum albumin
DAPI	4′,6-Diamidino-2-phenylindole dihydrochloride
DCF-DA	2′,7′-Dichlorodihydrofluorescein diacetate
ER	Endoplasmic reticulum
ERK	Extracellular signal-regulated kinases
JNK	c-Jun N-terminal kinase
MAPK	Mitogen-activated protein kinases
MTT	3-(4,5-Dimethyl-thiazol-2-yl)-2,5-diphenyl-etrazolium bromide
NAC	*N*-acetyl-l-cystein
PARP	Poly (ADP-ribose) polymerase
PI	Propidium iodide
PVDF	Polyvinylidenefluoride
ROS	Reactive oxygen species
