# Supplementary material for: Inhibition of Autophagy Promotes Salinomycin-Induced Apoptosis via Reactive Oxygen Species-Mediated PI3K/AKT/mTOR and ERK/p38 MAPK-Dependent Signaling in Human Prostate Cancer Cells
Source: Int J Mol Sci. 2017 May 18;18(5):1088. doi: 10.3390/ijms18051088 (PMC5454997; doi:10.3390/ijms18051088)
Supplement: Supplementary file 1 [file ijms-18-01088-s001.pdf]

Inhibition of Autophagy Promotes Salinomycin-Induced Apoptosis via Reactive Oxygen Species-Mediated PI3K/AKT/mTOR and ERK/p38 MAPK-Dependent Signaling in Human Prostate Cancer Cells

Kwang-Youn Kim[1,2†], Kwang-Il Park[3†], Sang-Hun Kim[2], Sun-Nyoung Yu[2], Sul-Gi Park[2], Young Woo Kim[1], Young-Kyo Seo[4], Jin-Yeul Ma[3], Soon-Cheol Ahn[2,5*]

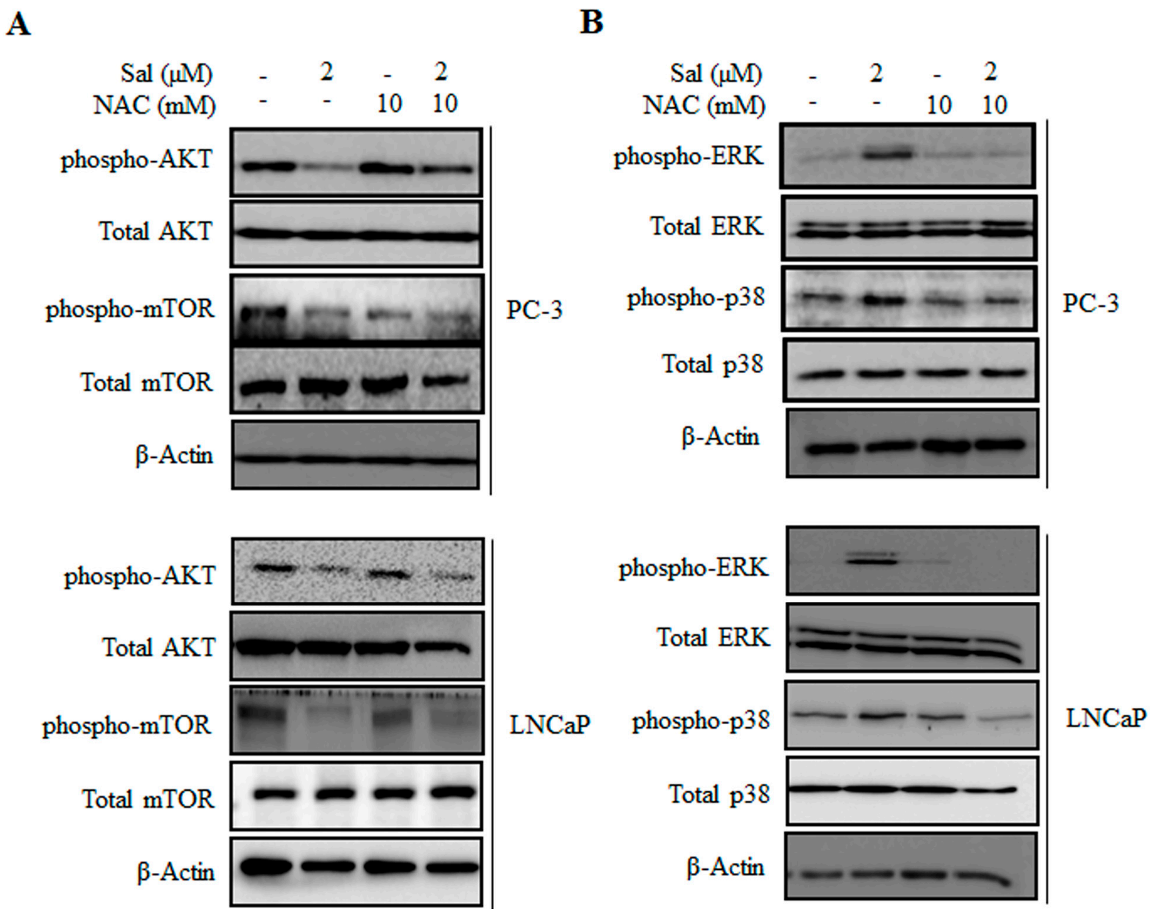

Supplementary Figure S1. ROS production regulates the PI3K/AKT/mTOR and ERK and p38 MAPK pathways. (**A**) Effects of NAC on AKT/mTOR protein expression; (**B**) Effects of NAC on ERK and p38 MAPK protein expression. Cells were pretreated with 10 mM NAC for 1 h, and then the proteins were detected by western blot analysis. All data are representative of at least three independent experiments. Data are shown as the mean ± SD. * $p < 0.05$.
